# High-Temperature Deformation Behavior and Microstructural Characterization of Ti-35421 Titanium Alloy

**DOI:** 10.3390/ma13163623

**Published:** 2020-08-16

**Authors:** Danying Zhou, Hua Gao, Yanhua Guo, Ying Wang, Yuecheng Dong, Zhenhua Dan, Hui Chang

**Affiliations:** 1 Tech Institute for Advanced Materials & College of Materials Science and Engineering, Nanjing Tech University, Nanjing 210009, China; zhoudanying@njtech.edu.cn (D.Z.); zhoudanying321@163.com (H.G.); guoyanhua@njtech.edu.cn (Y.G.); dongyuecheng@njtech.edu.cn (Y.D.); zhenhuadan@njtech.edu.cn (Z.D.); 2 Jiangsu Collaborative Innovation Center for Advanced Inorganic Function Composites, Nanjing Tech University, Nanjing 210009, China; 3 Chengdu Advanced Metal Materials Industrial Technology Research Institute Co. Ltd., Chengdu 610000, China; wangying2205b@163.com

**Keywords:** near-β titanium alloy, hot deformation behavior, α phase spheroidization, dynamic recovery, dynamic recrystallization

## Abstract

A self-designed Ti-35421 (Ti-3Al-5Mo-4Cr-2Zr-1Fe wt%) titanium alloy is a new type of low-cost high strength titanium alloy. In order to understand the hot deformation behavior of Ti-35421 alloy, isothermal compression tests were carried out under a deformation temperature range of 750–930 °C with a strain rate range of 0.01–10 s^−1^ in this study. Electron backscatter diffraction (EBSD) was used to characterize the microstructure prior to and post hot deformation. The results show that the stress–strain curves have obvious yielding behavior at a high strain rate (>0.1 s^−1^). As the deformation temperature increases and the strain rate decreases, the α phase content gradually decreases in the α + β phase region. Meanwhile, spheroidization and precipitation of α phase are prone to occur in the α + β phase region. From the EBSD analysis, the volume fraction of recrystallized grains was very low, so dynamic recovery (DRV) is the dominant deformation mechanism of Ti-35421 alloy. In addition to DRV, Ti-35421 alloy is more likely to occur in continuous dynamic recrystallization (CDRX) than discontinuous dynamic recrystallization (DDRX).

## 1. Introduction

Near-β titanium alloys have excellent mechanical properties and corrosion resistance, which are very suitable for large load-bearing material [1,2,3]. Therefore, such alloys are widely used in aerospace and marine engineering. At present, near-β titanium alloys are mainly Ti-Al-Mo-V-Cr series alloys [2] with high V content, such as Ti-55511 (Ti-5Al-5Mo-5V-1Cr-1Fe) [4], Ti-5553 (Ti-5Al-5Mo-5V-3Cr) [5], Ti-55531 (Ti-5Al-5Mo-5V-3Cr-1Zr) [6] and TB-19 (Ti-3Al-5Mo-5V-4Cr-2Zr) [7], etc. However, V is not only expensive but also causes environmental pollution problems during manufacturing. To solve this problem, great efforts have been made to replace V by other low-cost alloy elements while maintaining or even improving the strength, flexibility, and fracture toughness of the primary alloys. Fe is a strong β-stabilizing element with lower price than V, Ni, and Ti. Titanium alloys with added content of Fe have been reported, such as Ti-5Al-2.5Fe alloy, Ti-6Al-5Fe-0.05B-0.05C alloy [3,8,9,10,11]. They all reveal excellent mechanical properties. Ti-5Al-2.5Fe alloy exhibits equivalent rotating bending fatigue strength to Ti-6Al-4V ELI alloy. The highest strength of Ti-6Al-5Fe-0.05B-0.05C alloy can reach 1136 MPa. Besides, the addition of Fe can improve thermomechanical processing by reducing flow stress [9] and thus reducing the production costs as well. Therefore, Fe element is a promising alloying element that can reduce raw material and processing costs in the titanium alloy industry.

It is noteworthy that near-β titanium alloys are commonly used after isothermal forging and die forging. What is more, near-β titanium alloys are generally sensitive to processing parameters [1,6,8,12,13,14]. Hence, to further optimize the process and control the microstructure evolution during the crafting process, the influence of process parameters on the deformation characteristics of near-β titanium alloy need to be further understood [15]. Some investigations have been carried out on the hot deformation behavior, microstructural evolution, dynamic recovery (DRV), and dynamic recrystallization (DRX) of various near-β titanium alloys. For instance, Fan et al., [16] constructed deformation behavior and microstructure evolution of Ti-7333 alloy in the deformation temperature range of 770–970 °C and strain rate range of 0.001–10 s^−1^. They found deformation at higher temperature, and lower strain rate can form a more uniform microstructure. Korneva, Anna et al. Lv et al. [17] revealed that lower strain rate is beneficial to accelerate the fragmentation of acicular α phase inside Ti-55531 alloy. Zhao et al. [18] found discontinuous dynamic recrystallization (DDRX) and continuous dynamic recrystallization (CDRX) were the major microstructure evolution mechanisms of Ti-5553 alloy prepared by powder metallurgy and ingot metallurgy with different deformation conditions, respectively. The deformation activation energy is an indicator of the difficulty of hot compression on metals. It helps to determine the dominant deformation mechanism and the optimal processing window under different conditions. Meanwhile, Zhao et al. [18] calculated the apparent activation energy and the activation energy maps for the alloys at two different rates. The apparent activation energy of powder metallurgy alloys was 368.17 kJ·mol^−1^ and the apparent activation energy of ingot metallurgy alloys was 447.16 kJ·mol^−1^. Hu et al. [19] demonstrated the dependence of the fraction of the spheroidized α phase and the DRX of β grains on the hot forming parameters of Ti-55511 titanium alloy. They indicated that α phases prevented dislocation tangles, and the DRX of β grains gradually increased with the reduced deformation temperature or the raised strain rate.

Recently, we reported a new near-β titanium alloy, Ti-3Al-5Mo-4Cr-2Zr-1Fe (Ti-35421, wt%) alloy developed near-β titanium alloy based on Ti-3Al-5Mo-5V-4Cr-2Zr (TB-19, wt%). The chemical composition of Ti-35421 alloy is to replace 5 wt% V in TB-19 with 1 wt% Fe while the total Mo equivalent stays the same for Ti-35421 alloy. Ti-35421 alloy has a high strength of 900–1450 Mpa, and medium elongation 6–15% [20]. As the new alloy has just been designed, the workability is still missing. In order to understand the hot workability of Ti-35421 alloy, high-temperature compression tests over wide ranges of deformation temperature and strain rate were carried out. Based on the experimental results, the work hardening behavior and flow softening behavior under different deformation temperatures and strain rates are discussed in detail.

## 2. Experimental Details

### 2.1. Experimental Sample

The experimental materials used in this work included titanium sponge (purity 99.8 wt%), aluminum molybdenum alloy (purity 99.9 wt%), industrial pure aluminum (purity 99.7 wt%), electrolytic chromium (purity 99.9 wt%), industrial pure iron (purity 99.8 wt%) and zirconium sponge (purity 99.9 wt%). A raw ingot of 10 kg was prepared approximately at 1700 °C with a Vacuum Arc Remelting furnace (Summit Levitation, Shenzhen, China). The melting process was repeated three times to obtain a homogeneous composition of the alloy. The forging process could be divided into two steps. The ingot was first heated to 1050 °C in a resistance furnace and kept for 1 h to ensure that the inside of the sample was fully heated. The overall deformation reached more than 60% for the first step and an as-forged part that cross section was 80 mm× 80 mm as-forged part was obtained. For the second step of forging, the alloy was heated to 860 °C, held for 1 h, and the ingot was forged into a 70 mm × 40 mm thick plate as its final dimension.

It was confirmed by the metallographic method that the β transformation temperature of Ti-35421 was approximately 815 ± 5 °C. The chemical composition was measured by Inductively Coupled Plasma (Thermofisher, Inductively Coupled Plasma Mass Spectrometry, Waltham, MA, USA) and listed in Table 1. The alloy was annealed under the β phase region (860 °C, 30 min, air cooled) to ensure the stability of the microstructure and release internal stress brought by the forging process before hot pressing.

### 2.2. Experimental Procedure

After using the Φ 8 mm × 12 mm cylindrical samples cut from the heat-treated samples, the thermocouple was soldered to the surface of the sample before the sample was placed in the Gleeble-3800 simulator system to ensure the temperature of the sample during the entire high-temperature thermal deformation process. It was ensured that the unidirectional compression test was carried out in a vacuum of 10^−2^ Pa at the deformation temperatures of 750 °C, 775 °C, 800 °C, 830 °C, 880 °C, 930 °C and the strain rates were 0.01 s^−1^, 0.1 s^−1^, 1 s^−1^,10 s^−1^ with a deformation degree of 60% height reduction. The samples were heated to the deformation temperature at a rate of 10 °C/min and kept for 3 min before the deformation started to ensure uniform temperature inside the samples. During the compression process, a certain amount of friction was generated between the two round end faces of the sample and the compression head. When the example entered the equipment, graphite sheets need to be added on both sides of the sample to reduce friction.

### 2.3. Metallography and Analysis

The deformed specimens were sectioned parallel to the stress axis for the microstructure evolution. Surface etching was conducted using Kroll reagent. The composition of the etching solution was 2% hydrofluoric acid + 8% nitric acid + 90% distilled water mixture, and the etching time was 5–10 s. The microstructure was observed on an Optical Microscope (Zeiss, Axio Observer A1m, Jana, German) and Scanning Electron Microscope (SEM, FEI, Scios, Waltham, MA, USA). For electron backscatter diffraction (EBSD) examination, the samples were electro-polished with a solution of 5% perchloric acid, 65% methyl alcohol, and 30% butanol at 25 °C with a voltage of 25 V and the polishing time was 40 s. The EBSD observation was carried out on a 7800 F scanning electron microscope. The EBSD scans were performed using a step size of 1 μm, and the experimental data analyzed by Channel 5.0 software (Oxford Instruments, Abingdon, Oxfordshire, UK).

## 3. Results and Discussions

### 3.1. Initial Microstructure

**Figure 1:**
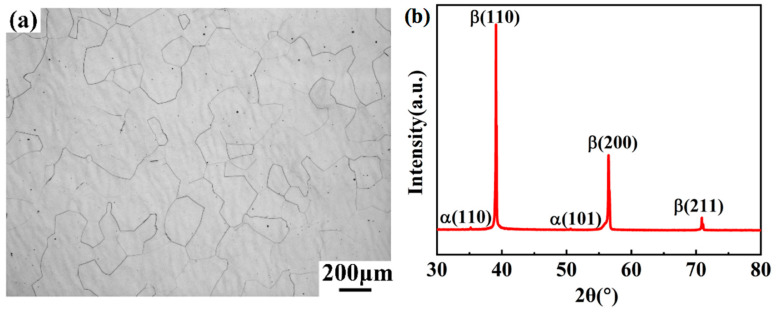
(**a**) Optical microstructure of the Ti-35421 alloy solution treated at 860 °C for 30 min and (**b**) its X-Ray diffraction pattern.

Figure 1a shows the air-cooled microstructure of the forged Ti-35421 alloy held at 860 °C for 30 min. Equiaxed β grains with a size between 200–300 μm were obtained. The XRD diffraction pattern of solution-treated Ti-35421 alloy is shown in Figure 1b. The pattern showed solution treatment is almost a full β phase with a body centered cubic (BCC)structure peak after solution treatment. The effect of elements on the stability of the beta phase for titanium alloys can be measured by the Mo equivalent equation [Mo] [21]. When [Mo]eq. is over 10, the high-temperature stable β phase of the titanium alloy can be completely maintained at room temperature after solution-treatment with rapid cooling. It can be concluded from Equation (1) [22] that [Mo] Equation of Ti-35421 alloy is 11.22, which indicates a full β phase with a bcc structure which could be obtained by a certain cooling procedure after solution treatment.

(1) [Mo=Mo+V/1.4+Cr/0.4+Nb/3.3+Fe/0.5+Mn/0.6+Ni/0.8+W/2+Ta/4+Co/0.9]

### 3.2. Flow Behavior

**Figure 2:**
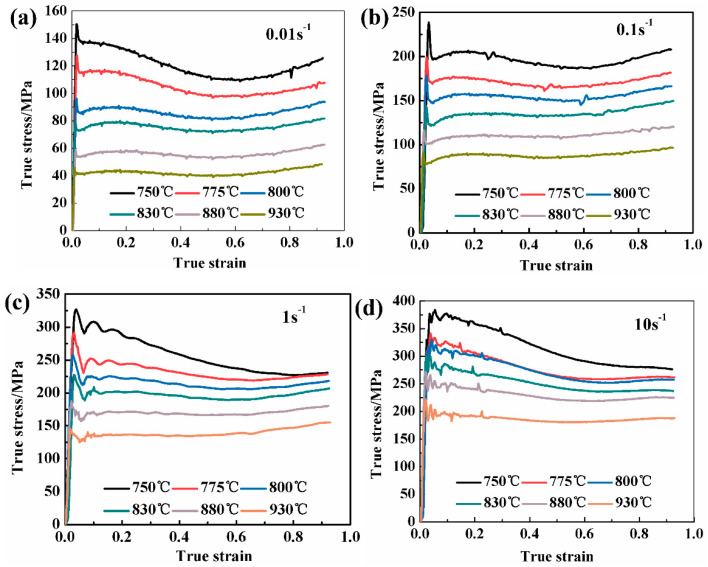
True stress–strain curves of Ti-35421 alloy at different strain rates (**a**) 0.01 s^−1^; (**b**) 0.1 s^−1^; (**c**) 1 s^−1^; (**d**) 10 s^−1^.

Figure 2 shows the true stress–strain curves at different deformation conditions. In all stress–strain curves, the increase in stress at the initial stage of the alloy is almost straight, which reflects the fact that the alloy has undergone a certain degree of work hardening. At this stage, as the strain increases, the dislocations continue to pile up, the dislocation density increases, and macroscopically there appears to be a rapid increase in the flow stress. After the true stress–strain curve reaches the peak value, the curve shows different shapes at different deformation rates with the further increase of strain, which can be divided into two characteristics: flow oscillation and flow softening. In the curves of various deformation rates, there are different degrees of discontinuous yield, that is, when the true stress reaches a peak, the stress drops sharply, and the up and down yield points appear in the stress–strain curve [23], as shown in Figure 2a,b. After the peak stress point, the stress–strain curves show a constant but not obvious yield phenomenon at strain rate of 0.01 s^−1^ and 0.1 s^−1^. However, when the strain rate is larger (>0.1 s^−1^), the curve shows an obvious yield phenomenon after reaching the peak stress point. In addition, the flow stress continues to drop after the end of the yield, indicating that the adiabatic temperature phenomenon or nonuniform deformation are obvious at 10 s^−1^. The continuous yield phenomenon in titanium alloy is mainly attributed to the reactivation of movable dislocations accumulated at grain boundaries. Grain boundary sliding (GBS) is generally the main deformation mechanism during the low strain rate forming process [24]. Kaibyshev et al. [25] found that the GBS mechanism can promote the transformation of Low angles grain boundaries (LAGBs) to high angles grain boundaries (HAGBs) and make the flow curve quickly enter the steady-state flow. Thus, it can be speculated that the flow stability of Ti-35421 alloy at a low strain rate is higher than that at a high strain shrinkage rate when the alloy deforms at the same deformation temperature.

Besides, the flow stress of Ti-35421 alloy increases on decreasing the deformation temperature and increasing the strain stress. When the strain rate increases or the deformation temperature decreases, the dislocation moves faster than the solute atoms that can be diffused, and it is challenging for the dislocation to be pinned by the solute atoms [26].

### 3.3. Apparent Constitutive Analysis

As a very classical constitutive model, the hyperbolic sinusoidal form of the Arrhenius equation (as shown in Equation (2)) is extensively used to express the relationship between flow stress, temperature and strain rate of alloys.

(2) $$έ=A{\left[\sinh\left(a\sigma \right)\right]}^{n}\exp\left(-Q/RT\right)$$

In Equation (2), έ is the strain rate (s^−1^), Q is the deformation activation energy (kJ/mol), R is the molar gas constant (takes 8.314 J·mol^−1^·K^−1^), σ is the flow stress (MPa), T is the absolute temperature (K) in deformation, A and α are temperature-independent material constants, and n is the stress value index. Due to Equation (2) being complicated, it can be simplified under certain conditions. For low-stress levels, when ασ < 0.8, the sinh (ασ) term can be reduced to an exponential form of stress:

(3) $$έ=A\sigma^{n_{1}}\exp\left(-Q/RT\right)$$

In the Equation (3), *n*_1_ is the temperature-independent stress index.

For higher stress levels, when ασ > 1.2, Equation (2) can be simplified as:

(4) έ=Aexp(βσ)exp(−Q/RT)

In Equation (4), β is a temperature-independent material constant. The parameter α can be estimated using the constants β and *n*_1_ according to Equation (5):

(5) $$\alpha =\beta /n_{1}$$

According to the hot compression test results of Ti-35421 alloys, the linear regression algorithm is used to calculate the coefficients in the constitutive equation. Taking the natural logarithm of both Equations (3) and (4) the following can be obtained:

(6) $$\ln έ=\mathrm{lnA}+n_{1}ln\sigma -Q/RT$$

(7) $$\ln έ={\mathrm{lnA}}_{2}+\beta \sigma -Q/RT$$

(8) $$n_{1}=\partial \ln\left(έ\right)/\partial \ln\sigma$$

(9) β=∂ln(έ)/∂σ

**Figure 3:**
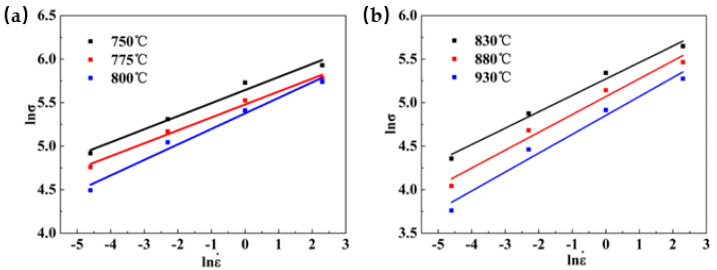
Relationship between lnσ and ln έ at different deformation temperatures: (**a**) α + β region; (**b**) β region.

**Figure 4:**
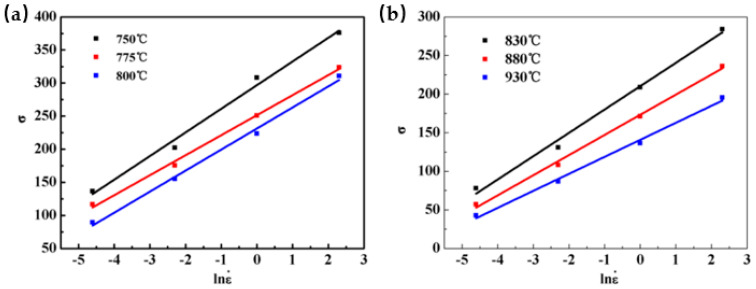
Relationship between σ and ln έ at different deformation temperatures: (**a**) α + β region; (**b**) β region.

Taking the strain as 0.1 as an example, the measured peak stress of Ti-35421 during hot deformation under different deformation temperatures is substituted into Equations (6) and (7), and the linear relationship curves of ln σ–ln έ and σ–ln έ are plotted, as Figure 3 and Figure 4 show. The linear relation of Figure 3 shows a good fit to each data point, and the average value of the slope of the line is used to find the n_1_ values of the α + β region and the β region, which are 6.33433 and 4.92614, respectively. The material constant β is calculated from Figure 4, and the average values are found to be 0.03082 and 0.11680, respectively. From Equation (5), the values of α are 0.00491 and 0.00799, respectively.

The following equation is obtained by rewriting Equation (2).

(10) lnέ=lnA+nln[sinh(ασ)]−Q/RT

If the strain rate έ is kept constant, a partial deviation between the two sides of Equation (9) is obtained, and Equation (11) is obtained.

(11) $$Q=R\;n\frac{\partial \ln\left[\sinh\left(\alpha \sigma \right)\right]}{\partial \left(1/T\right)}$$

**Figure 5:**
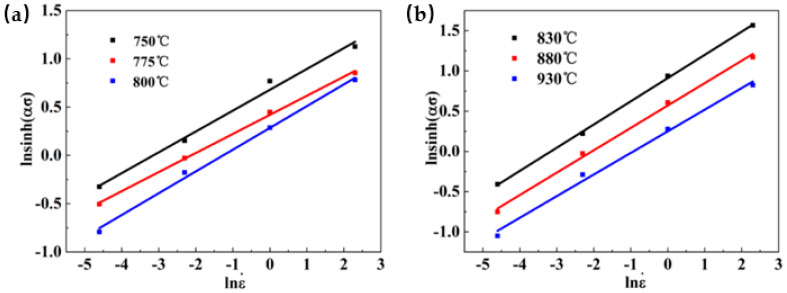
Relationship between ln[sinh (ασ)] and ln έ at different deformation temperatures: (**a**) α + β region; (**b**) β region.

**Figure 6:**
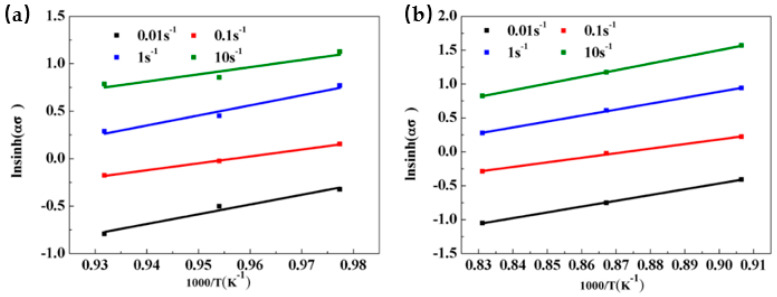
Relationship between lnsinh (ασ) and 1000/T at different deformation conditions: (**a**) α + β region; (**b**) β region.

Under constant temperature, the stress value index n can be determined by partial differentiation of Equation (10). Therefore, the value of n can be determined by the linear regression between ln[sinh (ασ)] and lnέ, as shown in Figure 5. The average calculated values of n in the α + β phase region and the β phase region are 4.70837 and 3.59496, respectively. According to Equation (11), to obtain the deformation activation energy Q, we must receive the partial derivative of ln[sinh (ασ)] to the inverse of the deformation temperature T. Figure 6 is the relationship of ln[sinh(ασ)]-1000/T, the slopes of ln[sinh(ασ)] to 1000/T are 8.9268 and 8.4753, respectively. The deformation activation energy of Ti-35421 titanium alloy can be obtained by introducing n, R, and the slope of ln[sinh(ασ)] to 1000/T in Equation (10). In the hot deformation process, the activation energy of hot deformation corresponds to the activation energy that controls the microscopic mechanism of hot deformation. The process needs to overcome the minimum barrier, which is the deformation activation energy (Q) during hot deformation.

**Figure 7:**
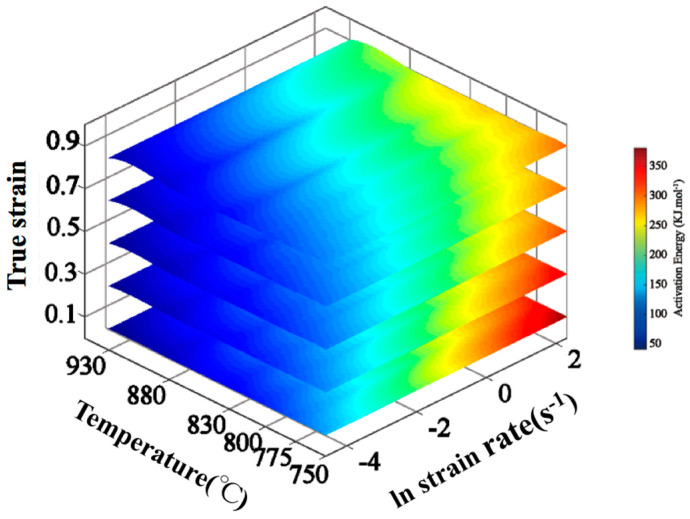
The 3D activation energy map of the Ti-35421 alloy obtained from modified hyperbolic sine mode.

In order to analyze the deformation mechanism of Ti-35421 alloy at hot deformation, a deformation activation energy diagram was constructed, as shown in Figure 7. The average deformation activation energies of Ti-35421 titanium alloy are 349.44 kJ/mol and 253.31 kJ/mol in the α+β and β phase region, respectively. The Q value of Ti-35421 titanium alloy is much higher than that of pure β-titanium (135–175 kJ/mol) [27] which indicates dynamic recovery (DRV) is the dominant hot deformation mechanism at the β phase region. In Figure 7, the lower the deformation temperature, the higher will be the strain activation energy needed. Moreover, at all deformation temperatures, the activation energy decreases when the strain exceeds the peak strain at an early stage and tends to be a constant value when the strain exceeds 0.5. Sudden changes in Q values can be explained by energy consumption mechanisms such as DRV and DRX. DRV and DRX act on the deformation by consuming the energy stored inside the alloy, making it easier for the sample to reach higher strains. Therefore, the deformation activation energy after the peak strain is significantly reduced.

### 3.4. Microstructural Evolution

The change of microstructure, flow curves, and activation energy of Ti-35421 alloy represents the change and transformation of the deformation mechanisms during the hot deformation process. Compared with the original microstructure (Figure 1a), the microstructure after hot deformation shows visible deformation characteristics. Generally, work hardening and flow softening are two competing processes that occur during metal thermal deformation, where flow softening is generally considered to be caused by deformation heating, strain localization, DRV, and DRX. These deformation mechanisms can be embodied in the stress–strain curve. As shown in Figure 2, the stress–strain curves of Ti-35421 alloy show different degrees of discontinuous yield. However, the alloy has a distinct softening mechanism in the α+β and β phase regions, and this is further discussed as follows.

#### 3.4.1. α + β Phase Region

**Figure 8:**
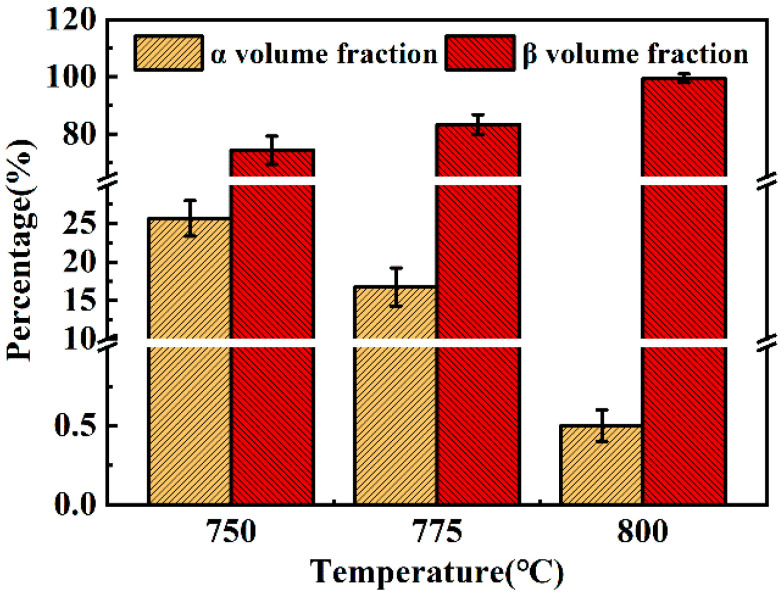
The variation of phase content with temperature at constant strain rate (0.01 s^−1^).

Figure 8 shows the variation of phase content with strain rate 0.01 s^−1^ as the deformation temperature ranges from 750 °C to 800 °C.The α and β phases can be identified by their morphology and contrast in the SEM image. The lighter phases are identified as β phase due to the higher atomic weight (Mo, Cr, Fe) concentration which causes more electron enrichment. Clearly, the volume fraction of α phases disappears gradually. The high deformation temperature can accelerate the element diffusion and atomic movement, which can accelerate the phase transformation [28]. Therefore, when the deformation temperature is 800 °C, almost no α phases can be found.

**Figure 9:**
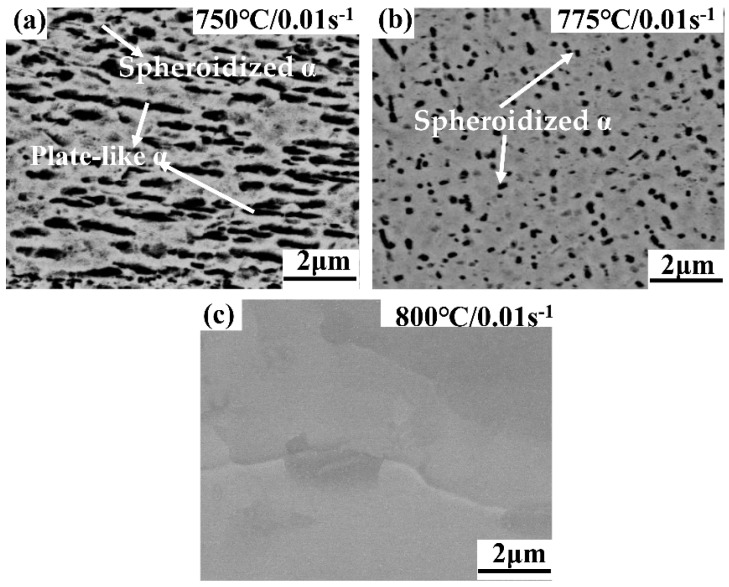
The size and morphology of α phases at different temperatures of α + β phase region: (**a**) 750 °C, 0.01 s^−1^; (**b**) 775 °C, 0.01 s^−1^; (**c**) 800 °C, 0.01 s^−1^.

Figure 9 depicts the size and morphology of α phases at the deformation temperature. The vertical direction of each graph is the compression direction. The samples deformed at 750 °C/0.01 s^−1^ (Figure 9a) show a large number of thick plate-like α phases and a few dense block-shaped α phases. Meanwhile, a lot of the lamellate α phases are fragmented and spheroidized at 775 °C (Figure 9b). This is because the grain boundary migration speed, element diffusion, and atomic motion are faster at high temperatures, and the spheroidization process is more complete [29]. Therefore, the fraction of spheroidized α phases obviously rises with the deformation temperature. However, almost all the α phases are transformed to β phases at 800 °C (Figure 9c). This means that 800 °C may be close to the β phase transition temperature. Meanwhile, the original structure is almost full β structure. Thus, when the deformation temperature is more than 800 °C, the main structure of the deformation is the β phase, and the α phase hardly exists.

**Figure 10:**
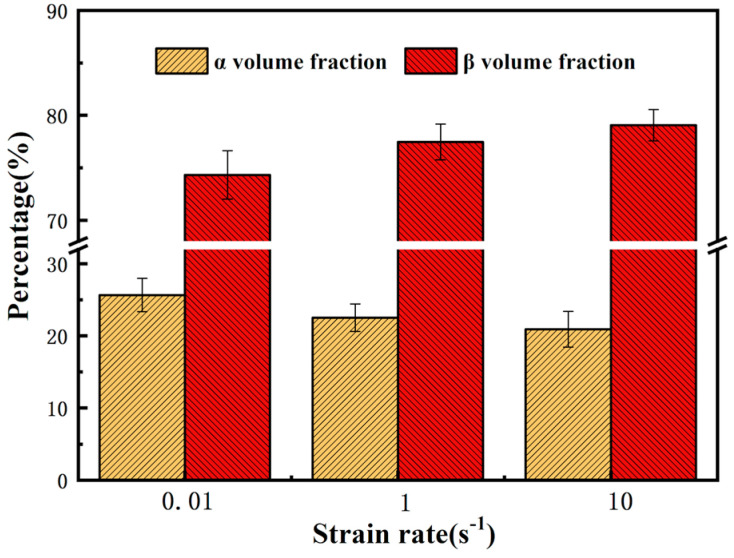
The variation of phase content with strain rate at constant temperature (750 °C).

Figure 10 shows the variations of phase content with deformation temperature at 750 °C when the strain rate changes from 0.01 s^−1^ to 10 s^−1^. The volume fraction of α phase is 25.68% at strain rate of 0.01 s^−1^, compared to 20.93% at a strain rate of 10 s^−1^. Dislocation entanglement becomes obvious with the increase of strain rate, which increases the dislocation density. Accumulation of dislocations can generate deformation energy stored inside the sample [30,31]. So hot deformation of high strain rate is beneficial for phase transformation. In addition, the temperature inside the samples continues to rise during the hot deformation process, especially at higher strain rates. Jia et al., [32] found that when metal is deformed at the strain rate of 10 s^−1^, the internal temperature of the metal will be 40 °C higher than the specified temperature. So, the actual deformation temperature is approaching beta transus. In this case, the volume fraction of α phases decreases.

**Figure 11:**
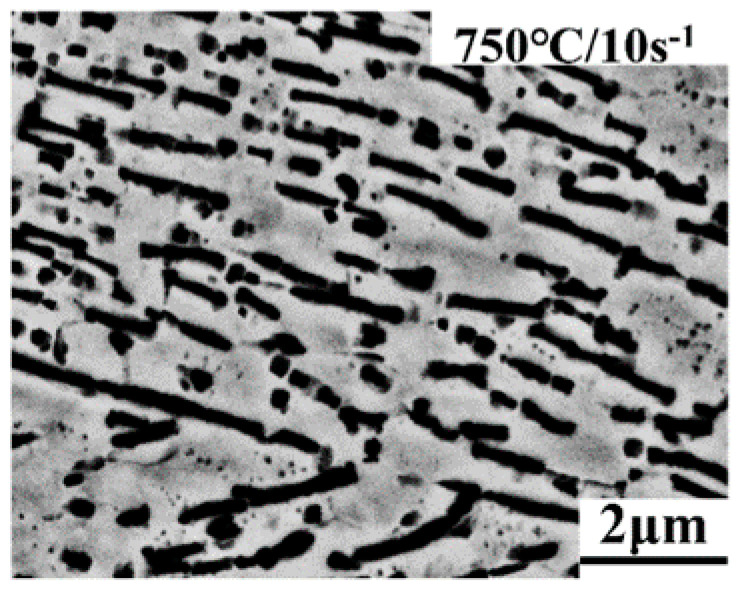
The size and morphology of α phases at deformation temperature of 750 °C and strain rate of 10 s^−1^.

The average size of plate-like alpha grains is 0.21 μm at strain rate of 10 s^−1^ and reduced to 0.17 μm at strain rate of 0.01 s^−1^. Therefore, the size of plate-like α grains declines with increasing strain rate, as shown in Figure 11. Furthermore, the lamellar α phase is almost not perpendicular to the deformation direction, and a few lamellate α grains are fragmented and spheroidized at 10 s^−1^. The high strain rate can reduce the volume fraction of the α phases, and there is not enough time to form α phases at high strain rates. Besides, only the α phases and β phases exist in Ti-35421 alloy. The β phases can spheroidize α grains by penetrating into the α phases [33]. Hence, a few small spherical α phases appear at high strain rates.

**Figure 12:**
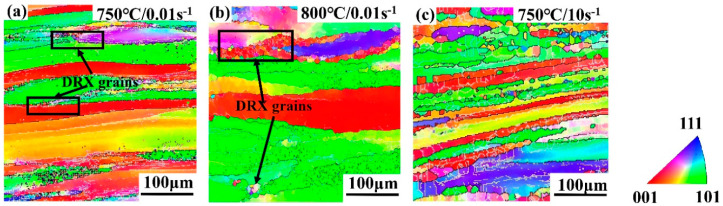
The EBSD analysis results of Ti-35421 alloy deformed at different conditions of α + β phase region: (**a**) 750 °C, 0.01 s^−1^; (**b**) 800 °C, 0.01 s^−1^; (**c**) 750 °C, 10 s^−1^.

**Figure 13:**
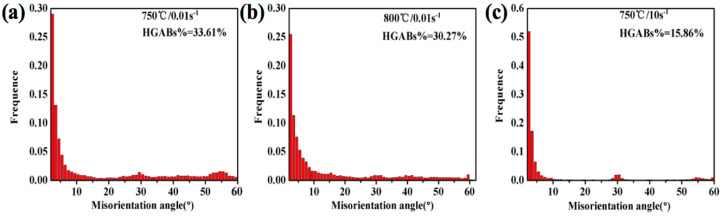
The relative fraction of low angles grain boundaries (LAGBs) and high angles grain boundaries (HAGBs) under different deformation conditions of α + β phase region: (**a**) 750 °C, 0.01 s^−1^; (**b**) 800 °C, 0.01 s^−1^; (**c**) 750 °C, 10 s^−1^.

Figure 12a,b shows the EBSD analysis of Ti-35421 alloys at 750 °C and 800 °C, when the strain rate is 0.01 s^−1^. Obviously, deformation temperatures have a great influence on microstructures. The samples deformed at 750 °C/0.01 s^−1^ (Figure 12a) show a homogeneous deformed microstructure and some fine DRX grains. On increasing the temperature to 800 °C (Figure 12b), grains are elongated into a serrated shape, and the size of DRX grains decreases. Meanwhile, the rose deformation temperature promotes the growth of β grains. This is because the grain boundary migration speed, element diffusion, and atomic motion are faster at high temperatures. Figure 13a,b shows that the misorientation angle distribution is sensitively affected by the deformation temperature. The fraction of HAGBs at 750 °C (33.61%) is more than that at 800 °C (30.27%). At the lower deformation temperature, the dislocation tangles are easy to form, which means that the DRX degree at 750 °C is more than that at 800 °C. However, at 800 °C, the DRV becomes the primary deformation mechanism, and the nucleation/rotation of substructures is dominant, which generates the disappearance of HAGBs. Therefore, the microstructure of Ti-35421 alloys consists of elongated serrated grains and α phase precipitates, which indicates the deformation mechanism is DRV and dynamic α globularization at 0.01 s^−1^ [18,34].

Figure 12c depicts the EBSD analysis of Ti-35421 alloys deformed at 750 °C and 10 s^−1^. Clearly, at 0.01 s^−1^, the elongated grain boundaries with a uniform LAGBs density inside, and recrystallized grains are distributed at the grain boundaries (Figure 12a,b). For 10 s^−1^, it can be found that elongated serrated grains have inhomogeneous deformation characteristics with low density LAGBs. The recrystallized grains are more than 0.01 s^−1^(Figure 12a), and the α phase is sparsely distributed in the grains (Figure 9b). These phenomena reveal that the deformation mechanism of high strain rate (10 s^−1^) consists of local deformation, DRX, and dynamic α precipitation.

#### 3.4.2. β Phase Region

**Figure 14:**
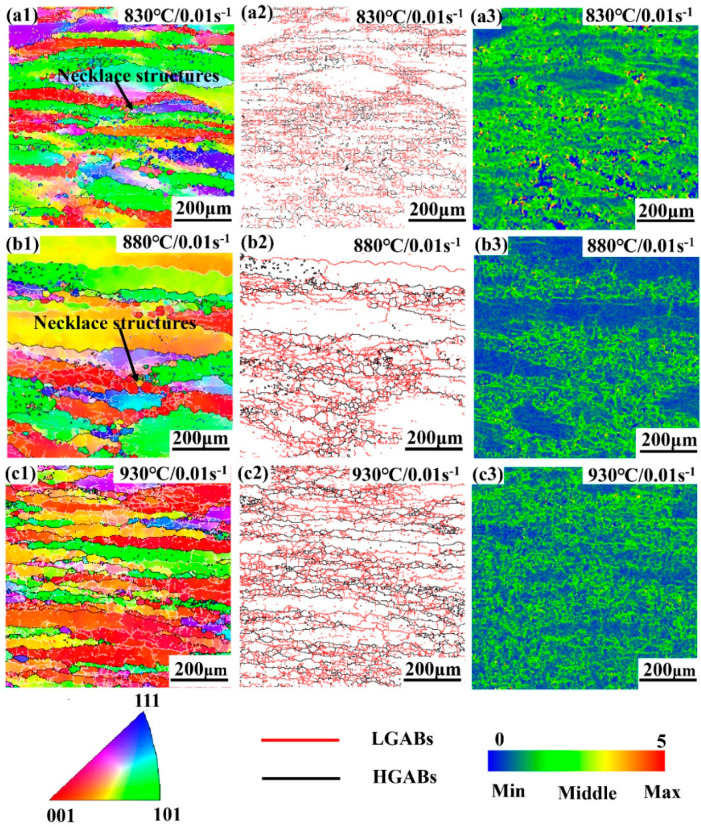
The EBSD analysis results of Ti-35421 alloy deformed at 0.01 s^−1^ under different temperatures of the β phase region: (**a**) 830 °C, 0.01 s^−1^, (**b**) 880 °C, 0.01 s^−1^, (**c**) 930 °C, 0.01 s^−1^ where the (1) is inverse pole figure, (2) is distribution of grain boundaries at high and low angle, and (3) is Kernel average misorientation under each condition.

**Figure 15:**
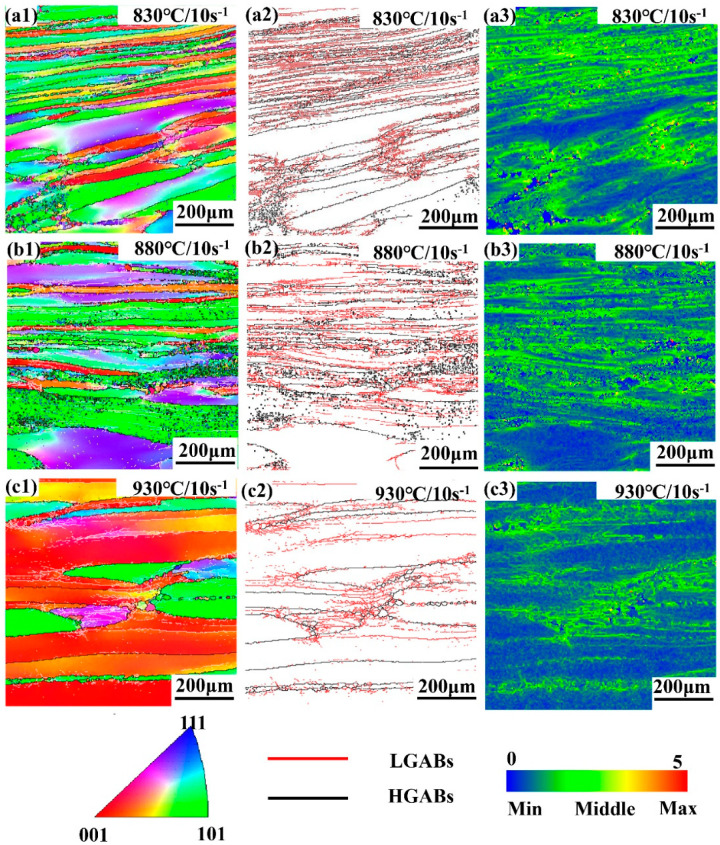
The EBSD analysis results of Ti-35421 alloy deformed at 10 s^−1^ under different temperatures of β phase region: (**a**) 830 °C, 10 s^−1^, (**b**) 880 °C, 10 s^−1^, (**c**) 930 °C, 10 s^−1^ where the (1) is inverse pole figure, (2) is distribution of grain boundaries at high and low angle, and (3) is Kernel average misorientation under each condition.

**Figure 16:**
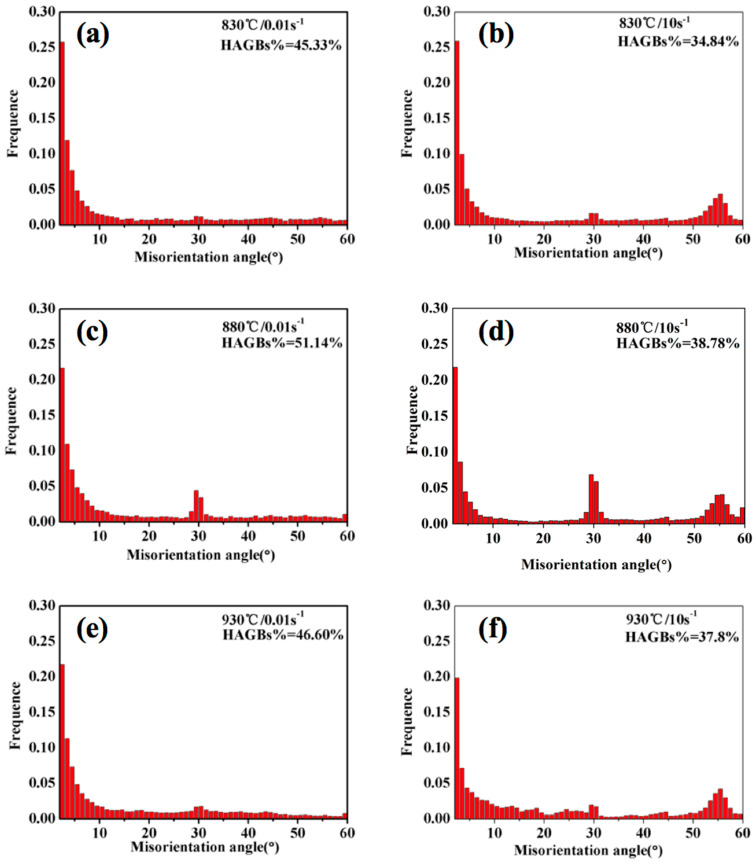
The relative fraction of LAGBs and HAGBs under different deformation conditions of the β phase region: (**a**) 830 °C, 0.01 s^−1^; (**b**) 880 °C, 0.01 s^−1^; (**c**) 930 °C, 0.01 s^−1^; (**d**) 830 °C, 10 s^−1^; (**e**) 880 °C, 10 s^−1^; (**f**) 930 °C, 10 s^−1^.

**Figure 17:**
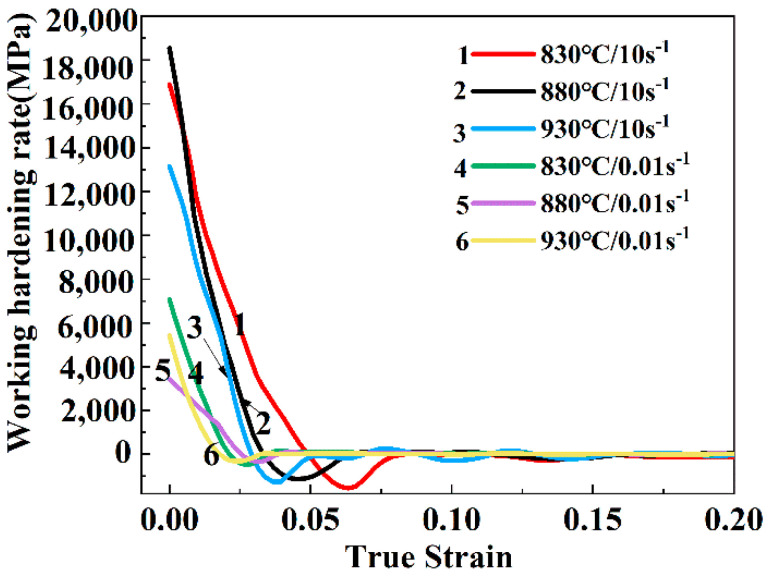
The curves of work hardening rates of Ti-35421 alloys at different deformation conditions.

Figure 14, Figure 15, Figure 16 and Figure 17 show the EBSD results of Ti-35421 alloy at different deformation conditions when the deformation temperature increases from 830 °C to 930 °C. From Figure 14 and Figure 15, it can be seen that there are fine grains under any deformation conditions, which suggests that Ti-35421 alloys always occur in DRX during the hot deformation of the β phase region. In addition, the volume fraction of DRX can be seen to be very low under any deformation conditions, so the main deformation mechanism of Ti-35421 is DRV.

Comparing Figure 14 and Figure 15, the deformation textures show obvious texture orientations, namely strong <100>, <101>, and weak <111>. When the strain rate is 0.01 s^−1^ and increasing the temperature from 830 °C to 930 °C, the <100> and <101> textures have different strengths, but the <111> orientation shows a weakening tendency. However, when the strain rate adds up to 10 s^−1^, the <100> orientation becomes strengthened gradually with the rise of deformation temperature.

When the strain rate is 0.01 s^−1^, there are coarse β grains with serrated boundaries and a minor number of equiaxed grains (Figure 14(a1–c1)), showing the characteristics of DRV and DRX. The equiaxed grains exist as “necklace structures” at the triple junction of the original grain boundaries, and this is a typical sign that CDRX has already nucleated [35,36]. It means that many recrystallized grains are distributed along the original grain boundary during deformation. On increasing the temperature to 930 °C, the equiaxed grains are coarser, as well as the β grains with serrated edges being more visible, which means there is a more intense DRV at this time. This phenomenon reflects a non-equilibrium state, DRV and DRX co-occur.

Meanwhile, the multiplication and rearrangement of dislocations seem to dominate at such a temperature and strain rate. Figure 14(a2–b2) shows the orientation map. Figure 16a–c depicts the fraction of boundaries according to the misorientation angle when the strain rate is 0.01 s^−1^. When the temperature is higher, the fraction of low-angle grain boundaries is lower. These reveal that the dominant mechanism is CDRX, the growth of DRX grains, and DRV at the strain rate 0.01 s^−1^. 

More interestingly, at 0.01 s^−1^, the fractions of the deformed grains and the sub-structured grains alternate calculated by the internal average misorientation angle of grains in Table 2. According to the internal average misorientation angle calculations, three types of microstructure can be identified. The grains with internal average misorientation angle above 1° are classified as deformed regions. If the grains consist of sub-grains whose internal average misorientation angle is under 1°, but the misorientation from sub-grain to sub-grain is above 1°, these grains are classified as substructured regions. All the remaining grains are classified as recrystallized regions. For the deformed sample at 0.01 s^−1^, the sub-structure grain fraction of 880 °C is higher than the value obtained at other temperatures. This suggests that the deformation of the Ti-35421 alloy at this time is sufficient to cause dislocations to multiply and entangle into sub-structured grains. When the temperature rises to 930 °C, it can be seen that the dynamically recrystallized grains enclose the substructure grains, and the grains are wavy, indicating that the accumulation and rearrangement of dislocations dominate at this temperature and strain rate [37]. It can also be seen from Figure 14(a3–c3) that the local orientation difference is abruptly increased with respect to KAM at 880 °C, which justifies this phenomenon.

Ti-35421 alloy at high strain rate (10 s^−1^) has a quite different microstructure compared to samples at low strain rate (0.01 s^−1^), as shown in Figure 15. There are obvious inhomogeneous deformation characteristics for this condition due to the short deformation time. Especially at 830 °C (Figure 15(a1)), only elongated grains are observed and no DRX grains are visible in the local deformation zone. With the increase of temperature, the phenomenon of inhomogeneous deformation characteristics gradually fades out. Meanwhile, the grains are elongated, and DRX grains are significantly less than at low strain rates. This indicates that the dominant mechanism is DRV in this case. Upon increasing the deformation temperature to 930 °C, some fine grains begin to form inside the original β grains due to CDRX. Observation of the microstructure shows that the hot deformation of Ti-35421 alloy is very sensitive to temperature at high strain rates (10 s^−1^). Figure 16d–f depicts the fraction of boundaries according to the misorientation angle when the strain rate is 10 s^−1^. Not only can we see that as the temperature increases, the proportion of high-angle grain boundaries increases, but also the fraction of HAGBs appears as small peaks around 60°. According to the formula of the angle between the crystal planes of bcc system, the angle between <111> and <101> is 61.55°, so twins are easily generated in the deformed structure of high strain rates (10 s^−1^).

The above results indicate that at higher temperatures and lower strain rates, hot deformation promotes material uniformity. From the microstructure observation of this work, it is evident that Ti- 35421 alloy is more prone to DRV during hot deformation.

### 3.5. Working Hardening and Dynamic Softening

The strain hardening rate θ can be used to analyze the relationship between work hardening and dynamic softening. It represents the slope of the stress–strain curve at a certain strain rate and deformation temperature, which can be expressed by Equation (12) [38,39]

(12) θ=dσdε

σ and ε represent the true stress and the true strain, respectively. When θ is positive, it means that the strain hardening rate increases with the increase of true strain, and this stage mainly occurs in work hardening. On the contrary, if θ is a negative value, it represents a dynamic softening at this time.

Figure 17 is a graph of the stress hardening rate of the Ti-35421 alloy as a function of deformation temperature and strain rate. It can be seen that at the primary stage of deformation, the value of θ is a positive value, which decreases sharply as the strain (less than 0.025) increases. This is due to the quenching and multiplication of dislocations, and the work hardening and dynamic softening alternately controls the flow behavior [38,39,40]. When the value of θ drops to around zero, it begins to decrease slowly and fluctuates around the zero lines. As θ becomes negative, the stress decreases gradually when the first dynamic softens, until θ decreases again to zero, corresponding to the first peak of Figure 2. At high strain rates, the gap between the strain hardening rate and the zero line is larger than at low strain rates, which implies the strain rate has a great impact on work hardening behavior. Also, in Figure 17, it can be seen that at the same deformation temperature, the higher the strain rate, the more pronounced is the flow softening. Generally, the inflection point can be seen as the incubation phase of DRX. As the strain increases, the dislocation density increases, and a substructure gradually forms [39]. Different from the low strain rate micro-mechanism, the DRV time is too short at high strain rates to limit the time of boundary migration, so that DRX nucleation through sub-grain aggregation and strain-induced boundary migration is delayed [10,26]. Therefore, DRX nucleation is under larger strain at the strain rate, as shown as Figure 15. In addition, the DRX nucleation rate is faster at high strain rates as the temperature increases. However, after that, at high strain rates, the time for DRX is limited because the stored thermal deformation is sufficient, so the accumulation of dislocations begins after the first yield until the second DRX occurs [37]. Therefore, the samples at high rate will undergo discontinuous softening, and the higher the temperature, the more obvious becomes the discontinuous softening effect, until the increase of dislocations and the quenching reach a balance, as shown in Figure 17. At low strain rates, there is no discontinuous softening of the sample since the deformation time is sufficient for DRX nucleation.

## 4. Conclusions

In this work, the rheological behavior and corresponding microstructure evolution of Ti-35421 alloy during hot deformation were studied. The conclusion can be made as follows:

1. The stress–strain curves of Ti-35421 alloy have obvious yielding behavior at a high strain rate. Mainly because of the sudden activation of the movable dislocation at the grain boundary.
2. The deformation activation energies of Ti-35421 alloys are 349.44 kJ/mol and 140.19 kJ/mol in the α + β and β phase region, respectively, based on the establishment of constitutive equations. In addition, as the temperature increases, more activation energy is required for hot deformation.
3. For the α + β phase region, dynamically α spheroidization and DRV is the dominant deformation mechanism at 0.01 s^−1^ strain rate, while the simple at 10 s^−1^ strain rate is controlled by local deformation and dynamical α precipitation. Lowering the deformation temperature leads to more precipitation of the α phase and coarsening.
4. Based on the observation of the microstructure and stress–strain curves of the β phase region, the volume fraction of the new recrystallized grains is less than 10%, which means that DRV runs through the hot deformation process. DRX is more prone to occur at low strain rates rather than high strain rates.
5. The samples at high strain rate undergo discontinuous softening, and the higher the temperature, the more obvious is the discontinuous softening effect.

## Figures and Tables

**Table 1:** Chemical composition of the as-received Ti-35421 alloy.

Element	Al	Mo	Cr	Fe	Zr	C	H	O	N	Ti
(wt%)	2.78	4.56	3.89	0.98	1.78	0.034	0.005	0.098	0.007	Balance

**Table 2:** Fractions of recrystallized, sub-structured, and deformed grains in the specimens deformed at different conditions of β phase region.

Temperature /°C	Strain Rate /S^−1^	Deformed Fraction /%	Sub-Structured Fraction /%	Recrystallized Fraction /%
830	0.01	75.92 ± 0.99	21.83 ±0.37	2.25 ± 0.02
830	10	88.83 ± 0.99	6.82 ± 0.32	4.35 ± 0.01
880	0.01	26.74 ± 0.24	71.38 ± 0.91	1.88 ± 0.01
880	10	89.06 ± 0.90	8.22 ± 0.097	2.72 ± 0.02
930	0.01	51.99 ± 0.39	45.01 ± 0.32	2.99 ± 0.02
930	10	13.25 ± 0.11	86.17 ± 0.23	0.57 ± 0.03
